# Electroacupuncture and Moxibustion-Like Stimulation Relieves Inflammatory Muscle Pain by Activating Local Distinct Layer Somatosensory Afferent Fibers

**DOI:** 10.3389/fnins.2021.695152

**Published:** 2021-07-15

**Authors:** Lizhen Chen, Xiaoyu Wang, Xiaoning Zhang, Hongye Wan, Yangshuai Su, Wei He, Yikuan Xie, Xianghong Jing

**Affiliations:** ^1^Institute of Acupuncture and Moxibustion, China Academy of Chinese Medical Sciences, Beijing, China; ^2^School of Basic Medicine, Peking Union Medical College, Institute of Basic Medical Sciences, Chinese Academy of Medical Sciences, Beijing, China

**Keywords:** electroacupuncture, moxibustion, A-fibers, C-fibers, inflammatory muscle pain

## Abstract

Recent studies have shown that both superficial and deep acupuncture produced clinically relevant and persistent effect on chronic pain, and several subtypes of somatic primary afferents played critical roles in acupuncture and moxibustion analgesia. However, which kind of primary afferents in the superficial and deep tissue of the acupoint is activated by acupuncture or moxibustion to relieve pain persistently remains unclear. The aim of this study is to investigate the roles of distinct peripheral afferents in different layers of the tissue (muscle or skin) in the acupoint for pain relief. Muscular A-fibers activated by deep electroacupuncture (dEA) with lower intensity (approximately 1 mA) persistently alleviated inflammatory muscle pain. Meanwhile, cutaneous C-nociceptors excited by noxious moxibustion-like stimulation (MS) and topical application of capsaicin (CAP) on local acupoint area produced durable analgesic effect. Additionally, spontaneous activity of C-fibers caused by muscular inflammation was also inhibited by dEA and CAP. Furthermore, decreases in pain behavior induced by dEA disappeared after deep A-fibers were demyelinated by cobra venom, whereas CAP failed to relieve pain following cutaneous denervation. Collectively, these results indicate that dEA and MS ameliorate inflammatory muscle pain through distinct primary afferents in different layers of somatic tissue; the former is achieved by activating muscular A-fibers, while the latter is mediated by activating cutaneous C-fibers.

## Introduction

Acupuncture is widely used to modulate chronic pain (Andersson and Lundeberg, 1995; Zhao, 2008). However, numerous high-quality randomized clinical trials (RCTs) of acupuncture analgesia obtained contradictory results. Recently, Vickers et al. (2018) analyzed the original data from 39 RCTs by an individual patient data meta-analysis and found that acupuncture was superior to no acupuncture for chronic pain with standard deviations (SDs) close to 0.5, while the SDs were close to 0.2 compared with the sham (including shallow needle penetration). The results suggested that effect sizes of acupuncture were associated with the type of control, which indicated that sham needle with superficial insertion in the skin was effective for pain and decreased the effect size of real acupuncture. Moreover, another RCT (Zucker et al., 2017) manifested that participants who had higher pain pressure thresholds had greater reduction in clinical pain following real acupuncture, while participants with lower pain pressure thresholds showed better analgesic response to sham acupuncture. Taken together, the evidence suggests that the analgesic effect is closely related to the depth and intensity of acupuncture.

Accumulated studies showed that acupuncture produced a promising analgesic effect *via* activating different primary fibers. Manual acupuncture (MA) can activate group I, II, III, and IV single afferent fibers in normal rats to relieve evoked pain (Kagitani et al., 2005, 2010; Huo et al., 2020). Moreover, emerging studies uncovered the interaction between A-fibers and C-fibers in the modulation of pathological pain. Stimulating deep nerve with A-fiber strength inhibited nociceptive C-neuron discharges to relieve acute neuropathic pain (Chen et al., 2019; Zhu et al., 2020). Also, continuous A-fiber threshold electric stimuli substantially decreased both antidromic C-fiber spontaneous activity and neurogenic inflammation in chronic neuropathic pain (Zhu et al., 2012). Furthermore, topical irritants such as capsaicin (CAP), mustard oil, and peppermint oil could attenuate inflammatory muscle pain by activating cutaneous C-fiber afferents (Fang et al., 2020). These studies shed light on different layers of somatotopic afferent nerves in relieving chronic pain. However, there is no accepted mechanism by which type of primary afferent fibers is activated by acupuncture and could have persisting effects on chronic pain, such as inflammatory muscle pain.

In this study, we attempted to demonstrate that the analgesic effect of activating non-nociceptive afferents in deep tissue by electroacupuncture (dEA) and cutaneous substance P or calcitonin gene-related peptide immune-reactive (SP-IR or CGRP-IR) fibers by moxibustion-like stimulation (MS). Distinct roles of somatosensory afferent nerves on pain relief were estimated by single-fiber recording in a muscular inflammatory pain model during dEA and MS, as determined in parallel experiments assessing spontaneous muscle pain. Following peripheral nerve blockade, the final experiments were designed to verify the specific peripheral afferents elicited by dEA or MS to relieve pain through inhibiting muscular C-fiber nociceptors.

## Materials and Methods

### Animals

Adult male Sprague–Dawley rats (200 ± 20 g) were provided by the Institute of Laboratory Animal Sciences, China Academy of Medical Sciences [experimental animal license number: SCXK(JING)2017−0005]. Rats were housed in a room temperature of 23°C under 12/12-h light/dark cycle-controlled conditions with free access to food and water. All experimental procedures were permitted by the Institutional Animal Welfare and Use Committee of the Institute of Acupuncture and Moxibustion, China Academy of Chinese Medical Sciences (D2020-09-25-1).

### Inflammatory Muscle Pain Model

Complete Freund’s adjuvant (CFA, #F5881, Sigma, United States; 200 μl) was injected into the right gastrocnemius under isoflurane inhalation anesthesia (0.5–1.5%) to induce inflammatory muscle pain. Control rats received an injection of equal volumes of saline.

### Electroacupuncture and Moxibustion-Like Stimulation

**FIGURE 1:**
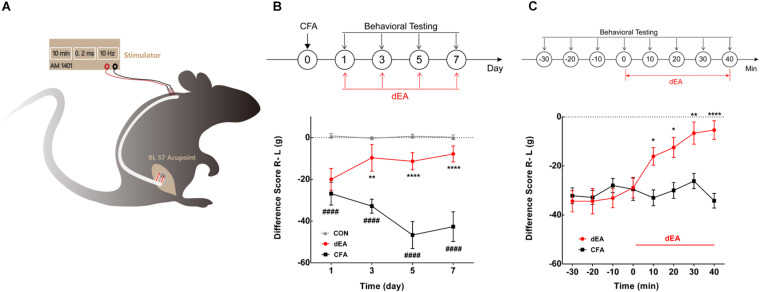
Deep electroacupuncture (dEA) ameliorated complete Freund’s adjuvant (CFA)-induced inflammatory muscle pain behavior. **(A)** Setup for dEA at the BL57 acupoint under free-moving condition. **(B)** The difference score for weight-bearing significantly reduced for 7 days after CFA injection and accumulated four times dEA markedly increased the difference score. ####*p* < 0.0001 vs. CON, **p < 0.01, or ****p < 0.0001 vs. CFA, significant difference between groups at each time point (Holm–Sidak post-test, *n* = 6). **(C)** The difference score for weight-bearing gradually increased and lasted during 40 min dEA stimulation. *p < 0.05, **p < 0.01, or ****p < 0.0001, significant difference between groups at each of the indicated time points (Holm–Sidak post-test, *n* = 8).

Acupuncture and MS were performed at right side BL57 (Chengshan) acupoint, which is located at the midpoint between the depression in the middle of the popliteal fossa and the depression posterior to the lateral malleolus of tibiofibula (Kim et al., 2017). Hair around the acupoints was shaved to expose the local skin before intervention. Superficial electroacupuncture (sEA) stimulation was applied with acupuncture needle (0.18 × 13 mm, Zhongyan Taihe, China) insertion into the subcutaneous tissue of BL57 acupoint at a depth of 2 mm for 10 min. Moreover, in order to only stimulate the muscle layer of the local acupoint and to conduct dynamic behavior monitoring under a free-moving condition, surgery was carried out to embed stimulation electrodes for dEA. Double silver wires wrapped with silica gel tube as the stimulation electrodes were embedded into the gastrocnemius at about 5-mm depth, which were led to the back of the neck through the loose connective tissue (Figure 1A). Animals recovered for 1 week before intervention. The electric stimulation parameter (10 Hz, 0.2 ms, 1 mA) output from the stimulator (AM1401 systems, United States) was used to induce muscle vibration.

Topical application of CAP (0.2%, 5 μl, Sigma, United States) or 50°C hot water (as noxious MS, for 10 min) on the local BL57 acupoint area was performed. For noxious MS, the bottom of a V-shaped glass tube was gently placed on the acupoint and 50°C water was injected through the V-shaped glass tube by a creep pump (LongerPump, BT300-2J, China). The 50°C water was provided from the thermostat water bath (HHS, Shanghai Medical Instrument Factory, Shanghai, China).

### Weight-Bearing Measurement

Weight-bearing measurement, a natural spontaneous pain indicator (Xie et al., 1995; Vermeirsch et al., 2007; Fang et al., 2020; Hill and Bautista, 2020), was carried out to observe inflammatory muscle pain behavior induced by CFA. Generally, rats were positioned with the hindlimbs on force plates in an Incapacitance Tester (Life Science, IITC600, United States). Rats were allowed to habituate the environment for 30 min before testing. The weight-bearing capacity (g) of each hindlimb was automatically averaged over a 5-s period. The difference score (g) was defined as weight-bearing on the right (experimental) hindlimb minus that on the left (control), which was determined as the average of three repetitions.

### Single-Fiber Recording

Rats were anesthetized with sodium pentobarbital [50 mg/kg, intraperitoneally (i.p.)]) and the rectal temperature was kept constantly around 37°C by a feedback-controlled heating blanket (DC, United States). Single-fiber recording was performed to detect the excitability of somatosensory afferent fibers as described previously (Hunt, 1954). Generally, the tibial nerve innervating the gastrocnemius or the cutaneous nerve over the gastrocnemius was exposed to the crus, covered by a pool of mineral oil. Firings of the single fibers recorded extracellularly by using silver wire electrodes were fed through an amplifier (AM-1800, A-M Systems, Sequim, WA, United States) with low-frequency filter at 1 Hz and high-frequency filter at 15 KHz. Signals were captured online and analyzed offline using the CED 1401-plus data acquisition system and the Spike 2 package (Cambridge Electronic Devices, Cambridge, United Kingdom). Single electrical pulses were delivered by a pair of bipolar silver stimulating electrodes distal to the recording site to measure the conduction velocity (CV). According to the CV and/or the response to the stretch of muscle, the recorded afferents were generally identified into C-fiber (CV < 1.5 m/s) or A-fiber (CV ≥ 1.5 m/s) (Duan et al., 2013).

### Selective Demyelination of Deep A-Fibers by Cobra Venom

#### Surgery for Intra-Tibial Nerve Injection of Cobra Venom

The tibial nerve was exposed at the middle of the thigh by blunt dissection through the biceps femora under general anesthesia. Cobra venom (0.15 mg/5 μl saline, Naja naja, Sigma, St. Louis, MO, United States) was slowly (1–2 min) injected underneath the epineurium using a Hamilton syringe connected to a 31G syringe tip through a polyethylene tube (PE-10/10, Warner Instruments) (Zhu et al., 2012). The needle was left in place for an additional 5 min before withdrawal in order to prevent leakage. An indication of a successful injection of cobra venom was a brief twitch in the muscle. Saline was injected into the tibial nerve as a control. The intervention was carried out 15 min after injection.

#### Transmission Electron Microscopy

Firstly, a segment of the tibial nerve including the site of venom injection was isolated and placed in 4% glutaraldehyde (diluted with 0.1 M sodium cacodylate buffer) and stored at 4°C for more than 2 h. The samples were then fixed in 1% osmium tetroxide for 2 h, removed from the fixative, and rinsed three times (5 min/time) with 0.1 M phosphate-buffered saline (PBS, pH 7.4). Secondly, the samples were dehydrated in a graduated series of acetone (50, 70, 95%; SINOPHARM, China), respectively, for 15 min, and then were dried twice in 100% acetone for 10 min. Thirdly, Spon 812 resin (ZB-S0060, SPI, United States) was used to mount the samples. The samples were placed in a 2:1 ratio of dried 100% acetone and Spon 812 resin, then in a 1:2 ratio, followed by 100% acetone to resin for 2 h, respectively. Next, the samples were placed in Spon 812 resin and cured in an oven at 36°C for 12 h, at 48°C for 12 h, and at 60°C for 24 h. Finally, the samples were sectioned into 70-nm-thick slices *via* using a Leica ultramicrotome (Leica, EM UC6, Germany). Cross-sectional slices of tibial nerves were placed onto a copper wire grid and then stained with 2% aqueous uranyl acetate for 30 min and finally lead citrate for 30 min. The copper wire grids containing tibial nerve sections were inserted into transmission electron microscope (H-7650, Hitachi, Japan). Two or three images were collected from each examined section to photograph.

### Denervation of the Skin Over the Gastrocnemius

Cutaneous denervation surgery was conducted 3 days before injection of CFA. Under isoflurane anesthesia, the cutaneous branches of the nerve over the gastrocnemius were bluntly separated and transected. After the surgery, the skin had no response to nociceptive mechanical stimuli of a pinch by tweezers, which indicated successful denervation (Xie et al., 1995). The sham group underwent the same procedure without cutting off cutaneous branches. Additionally, CAP was topically applied by surrounding the denervated skin, and SP/CGRP-IR fibers were used to characterize cutaneous C-fiber for validation of this surgery (Figures 5C–E).

### Immunohistochemistry

After stimulation, anesthetized rats (day 5 after CFA injection) were immediately perfused with 250 ml of saline, followed by 250 ml of cold 4% paraformaldehyde in 0.1 M PBS (pH 7.4). Skin tissue in the BL57 area (approximately 2 × 2 × 1 mm^3^, 9 mg) was dissected. The collected tissues were post-fixed in 4% paraformaldehyde at 4°C for 4 h and cryoprotected in 25% sucrose in 0.1 M PBS at 4°C for 24 h. After post-fixation, the skin was embedded in artificial medium (Shandon Cryomatrix, 120 ml, Thermo Fisher Scientific, United States), frozen, and cut into 20-μm sections on a cryostat (Thermo, Microm International FSE, Germany). The sections were then thaw-mounted on SuperFrost^®^ Plus slides (Thermo Fisher Scientific, United States) and allowed to dry. The sequentially mounted slides were prepared for immunohistochemical staining.

After an initial wash in 0.1 M PBS, the tissues were preincubated in a solution of 3% normal goat serum and 0.5% Triton X-100 (T9284, Sigma) in 0.1 M PBS for 30 min to block non-specific binding. The sections were then incubated with primary antibodies for 24 h at 4°C. The primary antibodies were rabbit polyclonal anti-SP antibody (1:1,000, Abcam) and mouse monoclonal anti-CGRP antibody (1:1,000, Abcam). After washing in 0.1 M PBS for 20 min, goat anti-rabbit Alexa Fluor 488 or goat anti-mouse Alexa Fluor 594 secondary antibody (1:1,000, Molecular Probes, Eugene, Oregon, United States) was used to visualize the corresponding primary antibodies. Following a final wash in 0.1 M PBS, slides were coverslipped with PBS–glycerol. The sections were examined using a laser scanning confocal microscope (FV1200, Olympus) equipped with a digital camera (DP70, Olympus). Fifteen randomized sections from each group were counted for the sum length of SP-IR and CGRP-IR fibers using microimaging software (cellSens Standard 1.11, Olympus). All immunohistochemistry procedures for each staining combination were performed at the same time to ensure staining consistency.

### Data Analysis

All data were processed with the statistical analysis software package GraphPad Prism 5.0 and expressed as the mean ± SEM. Differences between two groups were analyzed using Student’s *t*-test, and differences among multiple groups were analyzed using one-way analysis of variance (ANOVA) followed by Holm–Sidak post-test. Two-way ANOVA with Holm–Sidak post-test was applied to compare repeated measurements at different time points among groups. A statistically significant difference was defined as a two-sided *p* < 0.05.

## Results

### Deep Electroacupuncture With A-fiber Strength Ameliorated Complete Freund’s Adjuvant-Induced Muscular Inflammatory Pain Behavior

The effect of dEA with A-fiber strength (1 mA) on muscle pain was continuously observed at days 1, 3, 5, and 7 after CFA injection, and dEA was delivered at the same time points. The difference score of weight-bearing after CFA injection gradually decreased and lasted for 7 days (*p* < 0.0001; Figure 1B), indicating the aggravation of muscle pain over time. dEA obviously increased the difference score from the second stimulation at day 3 after CFA (*p* < 0.01; Figure 1B). The dEA was applied every other day, and the analgesia effect was sustained until days 5 and 7 (^****^p < 0.0001; Figure 1B) as well. The result indicated that dEA not only ameliorated CFA-induced muscular inflammatory pain but also prohibited the development of inflammatory pain. Furthermore, a more detailed measurement of pain behavioral changes was conducted at day 5 to dynamically observe the immediate analgesic effect of dEA. As shown in Figure 1C, dEA for 40 consecutive min could increasingly improve pain behavior. These data suggested that dEA with A-fiber strength produced a relatively long-term suppression of CFA-induced inflammatory pain.

### Deep Electroacupuncture Inhibited Spontaneous Activity of C-Fibers in the Inflamed Muscle to Reduce Pain by Activating Muscular A-Fiber Afferents

**FIGURE 2:**
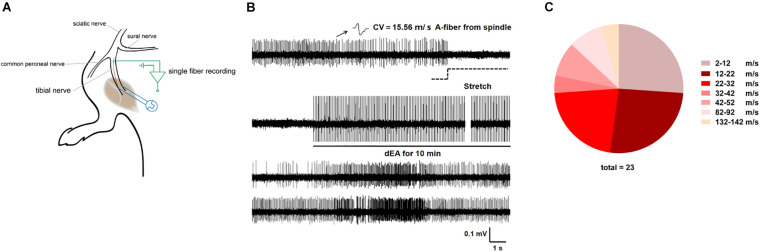
Deep electroacupuncture (dEA) activated numerous muscular A-fiber afferents from the gastrocnemius. **(A)** Schematic of *in vivo* recording for single fiber teased out from the tibial nerve innervating the gastrocnemius. **(B)** Representative single A-fiber [conduction velocity (CV) = 15.56 m/s] spontaneous activity originated from muscle spindle, and its robust discharges responded to muscular stretch and dEA. **(C)** Distribution of afferent fibers evoked by dEA according to CV. dEA activated numerous muscular A-fibers (26.96 ± 6.50 m/s, *n* = 23 fibers).

Single fibers innervating muscle spindle were isolated and recorded, subtypes of which were then categorized by their CV and mechano-responsiveness (Figure 2A). Figure 2B showed a representative firing trace of tonic muscle A-fiber afferents, and it was decreased by muscular stretch. However, 1 mA dEA, which was effective in reducing pain behavior as above, dramatically activated numerous A-fiber afferents (Figure 2C), indicating that muscular A-fibers might play a role in the neural mechanism of dEA analgesia.

**FIGURE 3:**
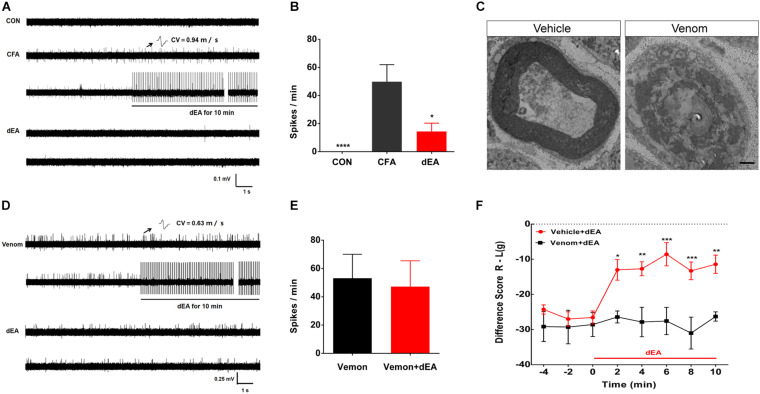
Deep electroacupuncture (dEA) reduced pain to inhibit C-fiber discharges by activating muscular A-fibers. **(A)** Representative traces showed that there was no C-fiber spontaneous activity in control rats, while complete Freund’s adjuvant (CFA) induced spontaneous activity of C-fibers [conduction velocity (CV) = 0.94 m/s]. The spontaneous activity of C-fiber was substantially inhibited by dEA. **(B)** Spikes of C-fiber were notably inhibited by dEA. *p < 0.05 or ****p < 0.0001 vs. CFA (*n* = 9 fibers). **(C)** Representative traces showed the demyelination of A-fiber axon after cobra venom injection into the tibial nerve (*n* = 2). The axon treated with saline had intact myelin sheath structure (*n* = 2). Scale bar, 1 μm. **(D)** Typical recordings of a single muscular C-fiber (CV = 0.63 m/s) had spontaneous activity after venom injection and had no response to dEA. **(E)** Spikes of C-fiber had no obvious response to dEA after venom injection (*n* = 10 fibers). **(F)** Venom completely blocked the effect of dEA on muscular pain. *p < 0.05, **p < 0.01, ***p < 0.001, significant difference between groups at each of the indicated time points (Holm–Sidak post-test, *n* = 7).

We next defined whether pain behavior was related to the abnormal activity of afferents from the tibial nerve innervating the inflamed gastrocnemius (Figure 2A). Lots of C-fibers discharged spontaneously in CFA-treated rats, while no spontaneous firing was observed in control rats (Figures 3A,B). Intriguingly, spontaneous activity of C-fibers sharply decreased, and only sparse discharges remained after dEA intervention (Figures 3A,B). The results suggested that dEA inhibited spontaneous activity of C-fibers derived from the inflamed muscle, which may be the underlying mechanism of the effect of dEA in relieving muscle pain.

In order to testify whether the inhibitory effect of dEA on C-fiber discharges was mediated by muscular A-fiber activation, cobra venom was used to pharmacologically block the myelinated fibers. Under the transmission electron microscope, we observed that venom caused an axon demyelination manifested as spiraling lamellae of the myelin sheath dissolved to the stray fragments and layers of myelin slackened (Figure 3C). After blocking A-fibers, dEA no longer caused the inhibitory effect on spontaneous activity of C-fibers (*p* > 0.05; Figures 3D,E) and pain behavior (Figure 3F). Collectively, these results demonstrated that dEA suppressed spontaneous activity of C-fibers to reduce muscle pain *via* the activation of muscular A-fibers.

### Noxious Moxibustion-Like Stimulation but Not Gentle Superficial Electroacupuncture Activated Cutaneous C-Fibers to Attenuate Muscle Pain

**FIGURE 4:**
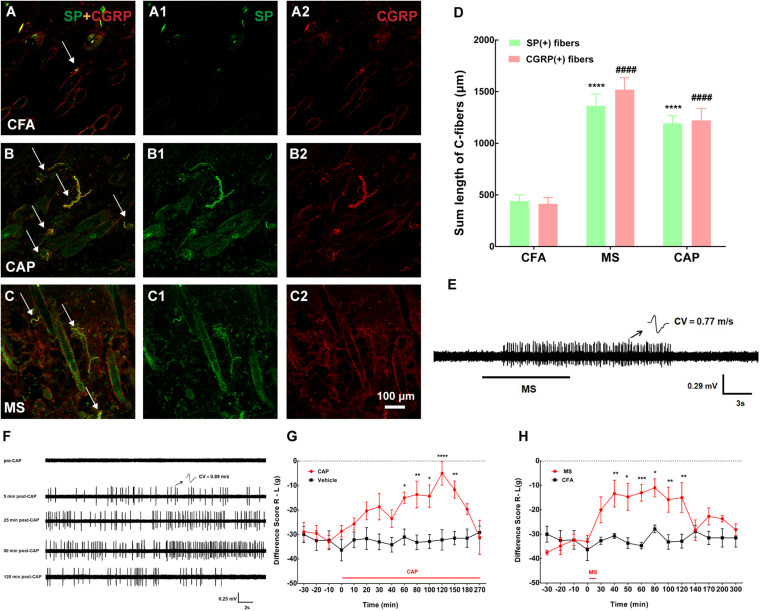
Both capsaicin (CAP) and moxibustion-like stimulation (MS) activated cutaneous C-fibers and substance P (SP)/calcitonin gene-related peptide (CGRP)-immune-reactive (IR) fibers and produced pain relief. **(A–C)** Double staining showed cutaneous SP-IR (green) or CGRP-IR (red) fibers in the BL57 in the three groups. MS or CAP intervention activated numerous SP/CGRP-IR fibers. (A1,A2,B1,B2,C1,C2) represented SP (A1,B1,C1 in green) and CGRP (A2,B2,C2 in red) in panels **(A–C)**, respectively. Double labeling of SP-IR and CGRP-IR fibers was in yellow (white arrow). **(D)** The summed lengths of SP/CGRP-IR fibers were longer after MS. SP: ****p < 0.0001 vs. complete Freund’s adjuvant (CFA); CGRP: ####*p* < 0.0001 vs. CFA (*n* = 15 sections selected randomly from 3 to 4 rats). **(E)** Representative traces of noxious MS activating cutaneous C-fiber afferents [conduction velocity (CV) = 0.77 m/s]. **(F)** Representative traces of CAP-evoked C-fiber firings. CAP application activated cutaneous C-fiber afferents (CV = 0.89 m/s) after 5 min and lasted for about 120 min. **(G,H)** The difference score of weight-bearing increased after MS and CAP application, respectively. *p < 0.05, **p < 0.01, ***P < 0.001, or **** *p* < 0.0001, significant difference between groups at each time point (Holm–Sidak post-test, *n* = 6).

Given the convincing role of muscular A-fibers in pain relief, the effect of cutaneous A-fibers by sEA (1 mA) on pain relief was examined. sEA could not relieve muscle pain (*p* > 0.05), indicating that the stimulation intensity of sEA was too gentle to achieve any analgesic effect. So, we topically applied CAP or noxious MS (50°C) on the local acupoint area to specifically activate cutaneous C-fibers and verify its influence on pain behavior. In particular, the effects lasted over 120 min (Figures 4G,H), which may be related to the relatively large stimulus intensity and area of CAP and noxious MS. In addition, both noxious MS and CAP application elicited discharges of the C-fibers in the skin (Figures 4E,F) and significantly increased the expressions of cutaneous SP-IR and CGRP-IR fibers (Figures 4A–D), which were not observed following sEA (data not shown). The results showed that noxious MS and CAP activated cutaneous C-fibers and attenuated muscle pain behavior, while gentle sEA did not cause any effect.

**FIGURE 5:**
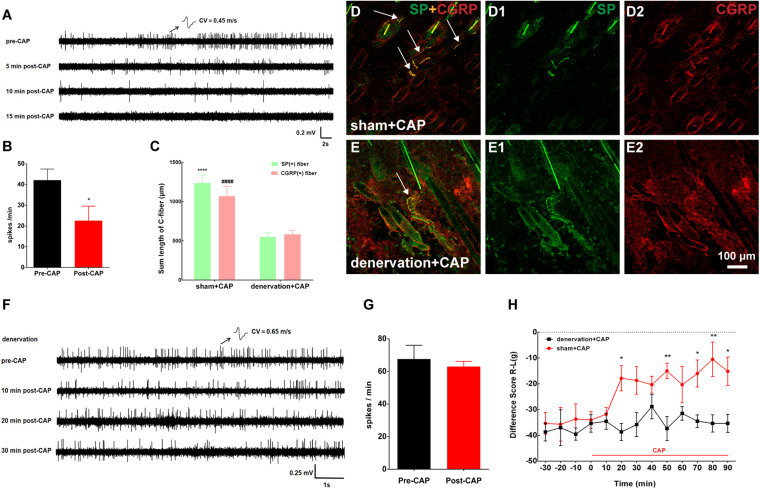
Topical application of capsaicin (CAP) on the local acupoint area suppressed nerve discharges of C-fibers to attenuate pain behavior by eliciting cutaneous C-fiber afferents. **(A)** Representative traces of C-fiber spontaneous activity inhibition [conduction velocity (CV) = 0.45 m/s] after CAP application. **(B)** Spikes of C-fiber were obviously inhibited by CAP. *p < 0.05 (*n* = 11 fibers). **(C)** The summed lengths of substance P (SP)/calcitonin gene-related peptide (CGRP)-immune-reactive (IR) fibers had significantly declined after CAP application under cutaneous denervation. SP: ****p < 0.0001 vs. denervation + CAP; CGRP: ####*p* < 0.0001 vs. denervation + CAP (*n* = 15 sections selected randomly from 3 to 4 rats). **(D,E)** Double staining was conducted to label C-fibers with SP-IR (green) or CGRP-IR (red) fibers in dermis of BL57 in the two groups (white arrow). **(F)** Representative traces of cutaneous denervation abolishing the inhibitory effect of CAP application on C-fiber spontaneous activity (CV = 0.65 m/s). **(G)** Spikes of C-fiber inhibited by CAP application nearly completely abolished by denervated nerve. *p* > 0.05 (*n* = 5 fibers). **(H)** Cutaneous denervation completely blocked the effect of CAP on pain behavior. *p < 0.05, **p < 0.01, significant difference between groups at each time point (Holm–Sidak post-test, *n* = 6).

### Topical Application of Capsaicin on the Local Acupoint Area Inhibited C-Fiber Spontaneous Activity From the Inflamed Muscle to Alleviate Pain by Activating Cutaneous C-Fibers

Only CAP was representatively chosen to test in this section because our results above showed that CAP and noxious MS likewise activated cutaneous C-fibers of the local acupoint to relieve muscle pain. Consistent with the behavioral results above, as shown in Figures 5A,B, spontaneous firings of C-fibers in the inflamed muscle were dramatically reduced within 10 min after CAP and merely disappeared 15 min after CAP. The denervation of local skin over the inflamed muscle was further carried out to confirm if the analgesic effect of CAP was mediated by activating cutaneous afferents. Three days after cutaneous denervation, CAP-induced increase in cutaneous SP/CGRP expression remarkably declined (Figures 5C–E). At the same time, cutaneous denervation almost completely abolished the inhibitory effect of CAP on C-fiber discharges and pain behavior (Figures 5F–H). Taken together, these data demonstrated that CAP-induced suppression of spontaneous C-fibers and muscle pain behavior was mediated by activating cutaneous C-fibers.

## Discussion

**FIGURE 6:**
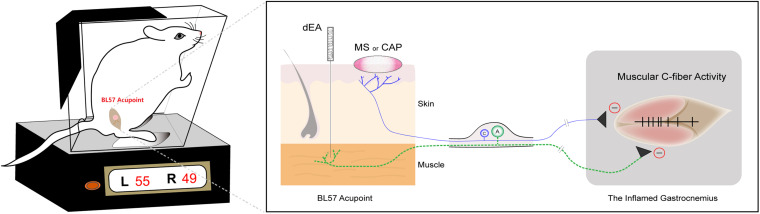
Diagram of distinct primary afferent pathways of deep electroacupuncture (dEA)- and moxibustion-like stimulation (MS)-induced analgesic effects. dEA activates muscular A-fibers at the BL57 acupoint, while noxious MS or capsaicin (CAP) excites cutaneous C-fibers to improve muscle pain behavior *via* inhibiting spontaneous activity of muscular C-fibers.

In the present study, we demonstrated that innocuous dEA with A-fiber strength in the muscle, noxious MS and CAP application on the skin produced persistent pain relief of CFA-induced inflammatory muscle pain in rats. Specifically, dEA activated muscular A-fibers, while noxious MS and CAP recruited cutaneous C-fibers to alleviate pain behavior *via* inhibiting spontaneous activity of C-fibers in the inflamed muscle. Consistently, these antinociceptive effects of dEA and CAP were abolished by deep A-fiber demyelination and skin denervation, respectively. The results elucidated that the analgesic effects of acupuncture and moxibustion partially depended on distinct somatic afferents in different tissue layers (Figure 6).

The kind of somatic afferents driven by acupuncture and moxibustion is critical to analgesia. Clinical studies reported that deep MA outperformed superficial MA in patients with myofascial pain (Ceccherelli et al., 2002). Moreover, a study also showed that locally transcutaneous electrical nerve stimulation activated joint afferents, but not cutaneous nerves, to generate anti-hyperalgesia in a rat model of inflamed knee joint (Radhakrishnan and Sluka, 2005). These results suggest that deep tissue afferents might be pivotal in acupuncture analgesia. The present study precisely testifies that dEA activates numerous muscle A-fiber afferents to relieve muscle pain.

On the other hand, cutaneous C-fiber afferents mediate the MS effect. Our previous study revealed that noxious MS (> 43°C) was able to drive long-distance neural reflex by activating peptidergic C-fibers (Su et al., 2015), which was in line with the results that noxious MS (about 50°C) activated local SP-IR or CGRP-IR cutaneous terminals in the present study. In addition, cutaneous nerve transection abolished the analgesic effect of C-afferents in the skin over the inflamed muscle (Xie et al., 1995; Fang et al., 2020). In this study, cutaneous nociceptor-mediated noxious MS analgesia was also demonstrated, while it disappeared after skin nerve transection. This result indicates that cutaneous nociceptive nerves play an important role during MS analgesia. Based on the above analysis, we conclude that distinct depth and afferent subtypes are vital determinants of EA and MS analgesia.

It has been identified that acupuncture alleviated pain behavior by releasing local analgesic substances, such as endogenous anandamide (Chen et al., 2009) and adenosine (Goldman et al., 2010). However, there is lack of neurophysiological evidence on these substances during local analgesia, especially explaining the mechanism underlying acupuncture stimulation with different parameters. The present study provides a solid proof that dEA activates muscular A-fibers and MS elicits cutaneous C-fibers to inhibit local C-fiber inputs to relieve chronic pain. Our results strongly support the analgesic effects of acupuncture in clinical practice with superficial and deep stimulation and highlight the precise roles of different afferent subtypes in superficial and deep layers in acupuncture analgesia, respectively.

In addition, the segmental acupuncture analgesia is generally explained by the gate control theory, in which acupuncture evokes homotopic A-fiber afferents to activate inhibitory interneurons in the spinal dorsal horn and subsequently prevent C-fiber inputs to the brain (Melzack and Wall, 1965; Xu et al., 2003; Zhu et al., 2004). However, the somatotopic layers where these A-fibers distribute have not been clearly characterized. Morphological studies proved that there were high-density myelinated nerve fibers innervating muscle (Sakita et al., 2020), which were mainly responsible for transmitting proprioceptive and positional sensation to nuclei of funiculi gracilis and cuneate (Hoheisel et al., 1989). It was also reported that spinal cord stimulation reduced neuropathic pain by activating deep A-fiber afferents in the dorsal column (Guan et al., 2010). Our study specifically shows that dEA activates numerous muscular A-fiber afferents to inhibit local C-fiber inputs and to attenuate pain, while this effect almost disappears after deep A-fiber demyelination. Intriguingly, we also observed that noxious MS exerts an analgesic effect by activating a large area of cutaneous peptidergic C-fibers, while sEA with A-fiber strength does not. It can be inferred that the larger area of noxious MS recruits more cutaneous C-fibers with a sum effect, indicating that the strength of EA and MS greatly relates to peripheral analgesia. For C-fiber-mediated acupuncture analgesia, studies showed that the more C-fiber inputs elicited by noxious stimuli, the stronger inhibition of noxious discharges of medullary subnucleus reticularis dorsalis (SRD) neurons (Xu et al., 2003), confirming that SRD neurons could be activated by the spatial summation of peripheral C-fiber inputs (Jin et al., 1978). Nociceptive transmission could likewise trigger the descending inhibition system to regulate SRD neurons (Pinto et al., 2008). Besides, noxious stimuli on the contralateral side (Fang et al., 2020) or BL57 acupoint area (data not shown) over the inflamed muscle failed to relieve pain behavior, in turn implicating that presynaptic inhibition of C-fiber inputs may be involved. C-fiber-driven inhibition of C-fibers functioned as a feedforward mechanism, by which the homotypic afferents control sensory information flowing into the spinal cord (Zimmerman et al., 2019; Fernandes et al., 2020). Further investigation should be performed to elucidate whether the descending pain inhibitory system or presynaptic inhibition mediates noxious MS analgesia.

## Conclusion

In summary, dEA activates muscular A-fibers at the BL57 acupoint, while noxious MS excites cutaneous C-fibers to inhibit spontaneous activity of muscular C-fiber and ameliorate pain behavior in rats. Both the depth and strength of EA and MS are critical for their analgesic effects.

## Data Availability Statement

The original contributions presented in the study are included in the article/supplementary material, further inquiries can be directed to the corresponding author/s.

## Ethics Statement

The animal study was reviewed and approved by the Institutional Animal Welfare and Use Committee of the Institute of Acupuncture and Moxibustion, China Academy of Chinese Medical Sciences (D2020-09-25-1).

## Author Contributions

LC mainly performed the experiment. XW, XZ, HW, and YS gave help for the method of this study. WH, LC, and XJ drafted the manuscript. XJ, WH, and YX were responsible for the conception, design, and the implementation of the study. All authors reviewed and agreed to the publication.

## Conflict of Interest

The authors declare that the research was conducted in the absence of any commercial or financial relationships that could be construed as a potential conflict of interest.
